# Nomophobia and smartphone addiction amidst COVID-19 home confinement: the parallel mediating role of digital gaming and social media tools usage across secondary school students

**DOI:** 10.3389/fpsyg.2023.1175555

**Published:** 2023-05-16

**Authors:** Mehmet Kemal Aydin, Metin Kuş

**Affiliations:** ^1^Distance Education and Research Center, Hitit University, Çorum, Türkiye; ^2^Department of Physical Education and Sports, Faculty of Sports Sciences, Hitit University, Çorum, Türkiye

**Keywords:** digital gaming behavior, nomophobia, secondary school students, smartphone addiction, social media tools usage

## Abstract

**Introduction:**

With the inevitable technological boom enforced by the COVID-19 lockdowns and online emergency remote teaching practices, the prevalence of nomophobia (NMP) and smartphone addiction (SA) among adolescents has become a pressing issue, which has come under scrutiny. However, the impact of social media tools usage (SMTU) and digital gaming behavior (DGB) on these phenomena remains unclear since there is little research focusing on the complex interplay among these variables. Regarding this context, the present study aimed to explore the parallel mediating role of secondary school students’ SMTU and DGB in the relation between NMP and SA.

**Methods:**

In line with this aim, we employed a cross-sectional design with a critical case sampling strategy and collected data through an online survey from a total of 427 secondary school students in Istanbul in the 2021-2022 academic year. In order to test the parallel mediation model, we employed multiple linear regression models by utilizing PROCESS models with 5000 BC bootstrap samples and 95% CI.

**Results and discussion:**

Results illustrated that there was an increase in the prevalence of NMP and SA during the COVID-19 home confinement as consistent with the previous research. The results also indicated that among the multiple mediators, the mediating role of SMTU was significant in the relation between NMP and SA. This means NMP has direct and indirect significant impact on SA through SMTU. However, the mediating role of DGB was found nonsignificant in this relationship. Our results are robust and hold key contributions to both theoria and praxis in educational psychology research realm by disentangling the complex underlying mechanism between NMP, SMTU, DGB, and SA. On the practical side, our results provide insightful implications for school boards and researchers in the development of effective interventions.

## 1. Introduction

Digital technologies have become an integral part of the modern life, characterized by rapid technological advancements. Children, particularly school-age students, are susceptible to adopting new technologies into their daily lives (Männikkö et al., 2018). In conjunction with the technological boom, the COVID-19 pandemic has profoundly impacted global populations, resulting in home confinement and restricted activities to curb the virus’s spread (Nilsson et al., 2022). Thus, the pandemic has substantially altered human behavior patterns worldwide with individuals compelled to remain at home. This has led to an upsurge in excessive technology use including overuse of smartphones and the internet along with increased digital gaming behavior (DGB) and social media tools usage (SMTU). Additionally, social isolation, financial difficulties, substance use, and poor mental health conditions such as depression, anxiety, and stress have contributed to this trend (Masaeli and Farhadi, 2021). Despite its suitability as an indoor activity, the increased use of technology has potential drawbacks. Excessive gaming and SMTU are some of these drawbacks. In particular with the pandemic period, excessive technology use has caused behavioral disorders and technological addiction including addictive SMTU and DGB. Previous research illustrated that a set of behavioral symptoms or disorders that are linked to the overuse of technology and the isolation caused by the pandemic comprise of social isolation, anxiety, depression, and insomnia (Huang and Zhao, 2020; Qiu et al., 2020). In a similar vein, the pandemic has limited adolescent socialization opportunities, leading to a considerable increase in SMTU, which may exacerbate addictive tendencies and negatively impact the well-being of adolescents (Marengo et al., 2022).

Over the past two decades, the proliferation of smartphones has made them the most important social media and digital gaming tool, particularly among adolescents. However, this has increased young people’s susceptibility, leading to the emergence of nomophobia (NMP), or No Mobile Phone Phobia. NMP is a prevalent problem among younger generations and has become increasingly common more recently (Anshari et al., 2019). The concept of NMP can be explained by four basic components, such as *unable to communicate*, *lose connectedness*, *unable to access information* and *give up convenience* (Yildirim and Correia, 2015). Although smartphones provide convenience to students and teachers in the education process and offer the opportunity to access mobile learning, information resources, and educational materials promptly and on demand, they have potential downsides such as NMP (Almunawar et al., 2015). With an endeavor to address this problem, previous research aimed to explain the associations between NMP and SA (Tavolacci et al., 2015; Anshari et al., 2019; Durak, 2019; Semerci, 2019), NMP and social media addiction (Lin et al., 2021), SA and social media addiction (Marengo et al., 2022), DGB and SMTU (Paschke et al., 2021; Nilsson et al., 2022).

In regard to pandemic conditions, home confinement and the curfews implemented during the COVID-19 pandemic and the resulting online participation of students in their classes have exacerbated students’ smartphone addiction (Serra et al., 2021; Marengo et al., 2022). In a similar vein, during lockdowns, smartphones, which became a source of entertainment and pastime apart from educational purposes. Likewise, this has contributed to an increase in students’ NMP (Shahzad et al., 2021). Previous research findings illustrated that even before the pandemic, one out of every three students have a problem with NMP (Tavolacci et al., 2015). Research focusing on the pandemic period also acknowledged that the COVID-19 pandemic prevented students’ social interactions, thus led to increased addictive use of smartphones (Pasquale et al., 2021; Serra et al., 2021), gaming behavior (Balhara et al., 2020), excessive internet use (Iyer and Sharma, 2020; Pasquale et al., 2021). Regarding the pandemic context, few studies focused on the SMTU profiles of adolescents during the pandemic (Muzi et al., 2021; Brailovskaia and Margraf, 2022; Marengo et al., 2022). Although all these studies have provided some significant contributions into a clear understanding of ABCs (Antecedents, Behaviors and Consequences) of addictive use of smartphones before and during the COVID-19 period, still there is a need for further studies to explore the complex interplay between NMP, SMTU, DGB, and SA since there are conflicting results. For example, previous research studies have asserted that playing digital games can offer potential advantages, including cognitive, emotional, and social benefits (Granic et al., 2014) and alleviation of isolation feeling (Carras et al., 2017). Furthermore, during the COVID-19 pandemic, digital gaming was found to be a helpful tool for managing stress and mitigating the negative effects of public health measures like lockdowns, quarantines, and social distancing (Balhara et al., 2020). Additionally, in some cases, playing digital games may function as a coping mechanism for addressing personal shortcomings or difficulties such as a lack of social support, relationship issues, or dissatisfaction with one’s appearance (Griffiths and Beranuy, 2009).

In view of these conflicting results existing in the literature, there is a need for exploration of the complex interplay between adolescents’ NMP and SA, SMTU and DGB as well as the underlying mechanism in this relation. Given this context, the present study aimed to investigate the mediating role of secondary school students’ SMTU and DGB in the relationship between NMP and SA. The study employed a cross-sectional design with a critical case sampling strategy and collected data through an online survey of 427 students in a secondary school in Istanbul during the 2021–2022 academic year. In order to test the parallel mediation model, multiple linear regression models with PROCESS macro using 5,000 bias-corrected bootstrap samples and 95% confidence intervals were used. The study’s findings present some invaluable implications for a better understanding of the role of SMTU and DGB in the relation between NMP and SA across secondary school students. More specifically, the present study holds key contributions to the existing literature in some respects. First, it discloses the complex interplay between the concepts of SMTU, DGB, NMP and SA. Additionally, it enables an increase in public awareness on digital media and the pandemic which have brought about behavioral disorders and technological addiction on secondary school students. The study adds to a better understanding of the mediating role of SMTU and DGB in the relation between NMP and SA. This study further highlights psychological implications of SMTU and DGB on psychosocial well-being and mental health. It also provides sociological implications of growing public concern about addictive SMTU and DGB. Additionally, the present study also provides some empirical originality regarding the critical case sampling since it is particularly useful in explaining phenomena of interest and it may provide unique insights into the relationship between NMP, SMTU, DGB, and SA among adolescents. Thus, we hypothesized a parallel multiple mediator model assuming that NMP impacts SA both directly and indirectly through SMTU and DGB across secondary school students in a context of COVID-19 home confinement.

## 2. Theoretical background and hypotheses

### 2.1. Self-regulation theory

Social Cognitive Theory (SCT) provides an underpinning framework for Self-Regulation Theory (SRT). According to SCT individuals’ behavior is shaped by their cognitive processes, as well as the social and environmental contexts in which they live. SCT describes how individuals can use their cognitive processes to control their behavior and attain their lifelong goals (Bandura, 1986). SRT was adopted as a theoretical foundation in the present study. SRT is a psychological theory that explains how individuals actively manage their cognitive, emotional, and behavioral resources to achieve their goals in different situations. SRT proposes that individuals can self-regulate their behavior and emotions through a series of cognitive and emotional processes. The underpinning idea lies behind SRT is that human beings are self-regulating organism who can actively adapt to their environment and set themselves goals and strategies to achieve those goals (Bandura, 1986). The control theory approach to self-regulation explains how goal-directed behavior is self-regulated that enable individuals to balance their own behavior. Self-regulation is a dynamic process that involves ongoing monitoring of one’s thoughts, emotions, and behavior to attain desired outcomes (Carver and Scheier, 2011). Social cognitive perspective on self-regulation emphasizes the importance of metacognitive processes, such as goal setting, planning, and self-reflection. This perspective views self-regulation as a cyclical process that involves setting goals, monitoring progress, and adjusting strategies as needed to achieve desired outcomes. Self-monitoring, which involves observing one’s own behavior, thoughts, and emotions, is one of the key components of SRT. Through self-monitoring, individuals can identify when their behavior or thoughts are not aligned with their goals and make necessary adjustments. SRT emphasizes the significance of self-efficacy, which refers to one’s confidence in their ability to achieve objectives and complete tasks successfully. People with high levels of self-efficacy are more likely to take goal-oriented actions and persist through obstacles and setbacks (Bandura, 1986).

SRT refers to the process of translating personal goals into action, which involves planning and adapting one’s thoughts, feelings, and behaviors to achieve desired outcomes (Kitsantas et al., 2000; Carver and Scheier, 2011). SRT can result in the potential benefit of avoiding distractions (Kopetz et al., 2013). Additionally, previous research indicated that SRT has been used as foundation in effective management of addictive behaviors such as internet addiction (Dawe et al., 2004), SA (Van Deursen et al., 2015), and addictive SMTU (Kitsantas et al., 2000). On the other hand, Jeong et al. (2016) asserted that a lack of self-regulation increases the risk of addictive SMTU and SA. Furthermore, depleted self-regulation capacity can have detrimental effects on healthy coping mechanisms, thereby increasing the likelihood of addictive smartphone and SMTU (Dijkhuis et al., 2017). To develop self-regulating skills, individuals need to manage competing demands for resources and time as well as aiming to achieve expected and planned outcomes while avoiding addictive SMTU (Neal et al., 2017). Individuals who lack self-regulating skills are thus more susceptible to addictive SMTU and SA. Overall, there is an established body of literature that explores the associations between these factors (Kuss and Griffiths, 2012; Kuss et al., 2014; Andreassen, 2015; Yildirim and Correia, 2015; Masaeli and Farhadi, 2021; Paschke et al., 2021; Pasquale et al., 2021; Marengo et al., 2022; Nilsson et al., 2022).

### 2.2. The associations between nomophobia and smartphone addiction, nomophobia and social media tools usage, nomophobia and digital gaming behavior

NMP is the anxiety and fear experienced by individuals when they are separated from or unable to use their mobile devices. It is increasingly prevalent as individuals get more reliant on their smartphones for social media and digital gaming. It is a real phenomenon which significantly impairs mental health and well-being causing symptoms of anxiety and depression (King et al., 2013). NMP has been explained as a problem derived from the inappropriate use of smartphone such as SMTU and DGB, which can be detrimental for the well-being of the user when used abusively and excessively (Viner et al., 2020). Smartphones can be considered as widely used and rapidly adopted tools throughout the evolution of ICTs and adolescents are the early adopters of them (Serra et al., 2021). In fact, the most important social media and digital gaming tool is the smartphone which makes adolescents susceptible to addictive behavior (Pasquale et al., 2021; Serra et al., 2021; Marengo et al., 2022). As a result of this vulnerability, one of the prevalent problems that adolescents face is NMP, which has emerged especially amongst the younger generation and has become increasingly common in recent years (Yildirim and Correia, 2015).

Social media tools have increasingly gained popularity amongst adolescents. Adolescents perceive social media as safe places to express themselves freely (Shah et al., 2019). Excessive use of social media can result in negative outcomes, including reduced self-esteem, social isolation, social anxiety, and NMP (Bergagna and Tartaglia, 2018). The widespread problematic SMTU among adolescents, which has increased during the pandemic, has been found to be positively associated with anxiety and negatively associated with self-esteem. In addition, excessive social media use by adolescents has been linked to higher levels of social isolation and loneliness (Ciacchini et al., 2023). Likewise, DGB is the habits and actions individuals display when playing games in a virtual environment. DGB can be explained as the state of deep engagement (Jennett et al., 2008). Thus, some individuals may become addicted to gaming, leading to negative consequences in their lives. These consequences can be attributed to excessive DGB, preoccupation with gaming, and withdrawal symptoms when unable to play (Griffiths, 2010). Excessive DGB during COVID-19 might cause psychological problems such as loneliness and depression (Sundaray and Galimotu, 2020). Previous research has identified depression, anxiety, social phobias, and poorer school performance as potential outcomes of pathological gaming (Gentile et al., 2011), indicating the need for further research.

The use of smartphones, while providing certain benefits, also brings about a number of associated problems. One such problem is the phenomenon of NMP, which can be defined as the fear of being without mobile phone contact. Research illustrates that excessive use of smartphones for SMTU and DGB has been linked to the development of NMP (Sandeep, 2018). The widespread usage of smartphones among adolescents for the purposes of social media and digital gaming renders them susceptible to experiencing NMP (Lee, 2014). The manifestation of social, physiological, and physical symptoms can be attributed to the addiction to smartphones (Anshari et al., 2019). Although smartphones provide adolescents with constant connectivity to the outside world and offer a limitless platform for digital gaming and social media, this reliance may result in the development of addictive tendencies. Of these tendencies, the most significant one is the display of NMP and SA (Durak, 2019; Semerci, 2019).

Given this context, previous research has attempted to explain the association between SA and NMP (Anshari et al., 2019; Durak, 2019; Semerci, 2019). In addition to this reliance, adolescents are at risk of developing NMP, due to addictive behaviors related to SMTU and DGB (Lin et al., 2021). Thus, there is a complex interplay between NMP, SMTU, DGB (Ayar et al., 2018; Ortega-Barón et al., 2021). Based on the previous research, the following research hypotheses were posited:

*H1.* NMP is positively associated with SA.

*H2.1.* NMP is positively associated with SMTU.

*H2.2.* NMP is positively associated with DGB.

### 2.3. The associations between social media tools usage and smartphone addiction, digital gaming behavior and smartphone addiction

Smartphone addiction is characterized by compulsive smartphone use that disrupts individuals’ daily activities, such as their schooling and social interactions. Studies have shown that there is a positive correlation between the amount of time spent on smartphone use and the likelihood of developing smartphone addiction (Serra et al., 2021). Research also illustrated that DGB and SA significantly predict SMTU (Savcı and Aysan, 2017). Adolescents are more likely to use technology such as the social media, digital games, internet, and smartphones. These technologies can increase their susceptibility to developing technological addictions (Valkenburg and Peter, 2011). For many adolescents the issues that occur most frequently are SA, SMTU and DGB. They use digital games for the purposes of engaging in enjoyable activities that provide entertainment, pleasure, and mental stimulation. However, excessive usage of them can cause addiction (Yiğit and Günüç, 2020), resulting in digital gaming addiction in most cases. Digital gaming addiction refers to a condition where a person becomes unable to cease playing games for extended periods, linking the game to actual life, impeding the obligations imposed on them as a result of playing games, and prioritizing gaming over other pursuits (Horzum, 2011). Previous research has evidenced that individuals who play digital games may exhibit addictive behaviors (Grüsser et al., 2007; Griffiths, 2010; Kuss and Griffiths, 2012). Digital gaming has been found to have a significant impact on individuals in various ways. Studies have revealed that digital gaming is associated with reduced self-confidence (Jeong and Kim, 2011), increased anxiety (Caplan, 2007; Wei et al., 2012). Furthermore, digital gaming has been linked to several dimensions of psychophysical health, including sleep, fatigue, and concentration problems. Moreover, it has been associated with a higher likelihood of experiencing depression (Männikkö et al., 2018). These negative effects underscore the need for a balanced and moderate approach to digital gaming and the importance of promoting physical activity, social interaction, and other healthy behaviors.

Although DGB and SMTU differ in many aspects, the World Health Organization labels them as examples of excessive screen time (World Health Organization, 2019). Recently, there has been a growing scientific focus on examining how various types of screen activities, including DGB or SMTU, may impact health outcomes, school performance, physical activity, and social relationships (Reed et al., 2019). The impact of DGB on SA still remains unclear since there is little research. However, research illustrated that the lockdowns during the COVID-19 pandemic have led to an increase in problematic DGB (Balhara et al., 2020) and screen time use (Fernandes et al., 2020). These factors might have a contributing role in adolescents’ development of SA. Thus, in the light of above arguments, the following hypotheses were set:

*H3.1.* SMTU is positively associated with SA.

*H3.2.* DGB is positively associated with SA.

### 2.4. The mediating role of social media tools usage and digital gaming behavior on nomophobia and smartphone addiction

Social media has become an essential aspect of adolescents’ daily lives. The widespread usage of internet and social media among adolescents in OECD countries has reached on weekdays 2 hours after school while on weekends more than 3 hours per day (OECD, 2017). The increased dependence or addiction to social media platforms has raised concerns among parents, teachers, governments, and young individuals themselves. The excessive SMTU and DGB has been linked to heightened feelings of anxiety and depression, as well as disrupted sleep patterns (OECD, 2017). Given the continuing rapid adoption of digital gaming and SMTU among young people, it is essential to adopt a strategy that mitigates potential risks while still allowing for the substantial opportunities and benefits that these technologies offer (OECD, 2017). Using social media excessively is connected to lower sleep quality and a notable correlation has been observed between playing digital games in the evening and experiencing sleep deprivation (Billari et al., 2018). There is a connection between social media use and body image issues (Fardouly and Vartanian, 2016) as well as the development of disordered eating habits (Holland and Tiggemann, 2016).

Research studies have indicated increased DGB and addictive SMTU have been linked to adverse outcomes and perception of decreased well-being (Nilsson et al., 2022). There is also a connection amongst time spent on digital gaming and SMTU, NMP and negative emotional and psychological symptoms (Brunborg et al., 2014; Carey et al., 2021). Smartphones, which are the most important tools of social media and digital gaming, can cause NMP amongst adolescents (Yildirim and Correia, 2015). Individuals who exhibit high levels of dependency on their smartphones are likely to experience a greater incidence of NMP (Azadmanesh et al., 2016). Studies have shown that individuals who exhibit high levels of dependency on their smartphones experience intense anxiety when they are separated from their devices or lose them (Volkmer and Lermer, 2019).

Moreover, the phenomenon of NMP results in various symptoms including feelings of isolation or loneliness, depression, social anxiety disorder, obsessive compulsive disorder, and other psychological disorders (Gezgin et al., 2018). A previous research study revealed that social media addiction was linked to poorer mental health and academic performance among students, and this association was mediated by self-esteem (Hou et al., 2019). Another study found that greater severity of depression was associated with increased problematic smartphone use (PSU) and symptoms of internet gaming disorder. Fear of missing out (FoMO) mediated the relationship between depression and PSU, and internet gaming disorder symptoms partially mediated the relationship between FoMO and PSU (Yuan et al., 2021). Similarly, a recent study indicated that peer exclusion, emotional symptoms, and FoMO were positively correlated, and FoMO mediated the association between peer exclusion and emotional symptoms (Marengo et al., 2021). Additionally, another study found that smartphone addiction played a mediating role in the relationship between NMP and aggression (Nuri et al., 2021). A recent research study examined the potential mediating effects of social network use, internet gaming disorder, and pathological internet use on the relationship between loneliness and depression. The results showed that loneliness was a positive predictor of depression. Moreover, the study found that the two mediators, internet gaming disorder and social network are parallel mediators in this relationship (Wang et al., 2021).

Our literature review reveals that most studies testing mediation models have focused on various factors such as internet addiction and psychological maltreatment, social media addiction, mental health, academic performance, FoMO, depression, PSU, SA, and internet gaming disorder (Fazeli et al., 2020; Marengo et al., 2021; Nuri et al., 2021; Wang et al., 2021; Yuan et al., 2021). These results may resonate the parallel mediating role of DGB and SMTU on the relation between NMP and SA. However, we have found an empirical research gap that, to our knowledge, no previous research examines the mediating role of SMTU and DGB on the association between NMP and SA. Thus, the underlying mechanism in this association and the extent to which NMP and SA are mediated by SMTU and DGB remains unclear. This necessitates further inquiry into this research area and highlights the empirical originality of the present study. Therefore, based on the above arguments and previous research gaps, the following hypotheses were proposed to test the parallel mediating role of SMTU and DGB in the relation between NMP and SA:

*H4.1.* SMTU mediates the relation between NMP and SA.

*H4.2.* DGB mediates the relation between NMP and SA.

## 3. Materials and methods

### 3.1. Research model

**Figure 1:**
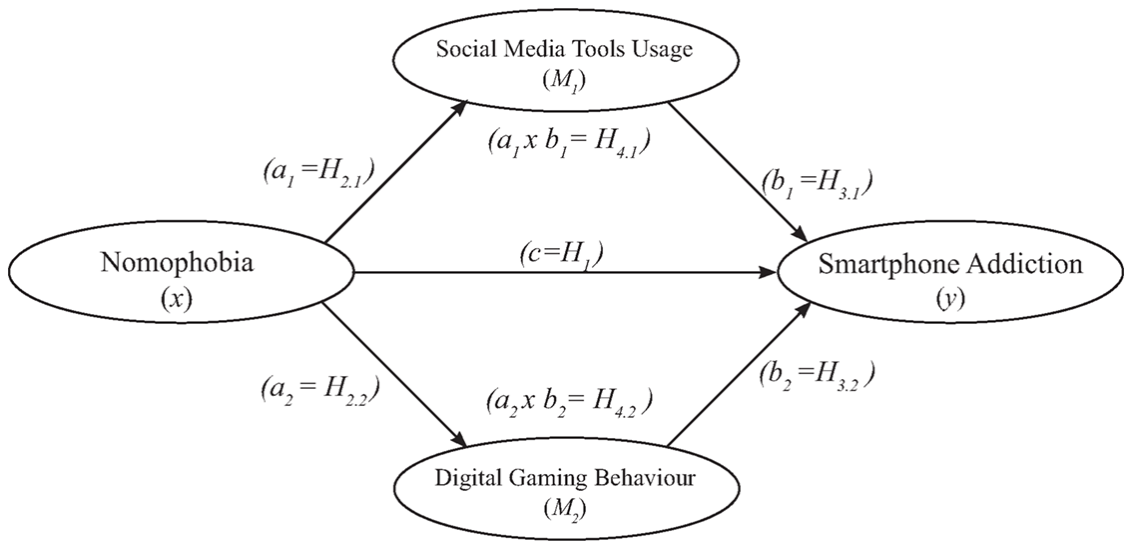
A conceptual model to test parallel multiple mediators’ impact on NMP and SA.

The present study employed a cross-sectional design with a multiple mediator approach in order to test parallel multiple mediator role of SMTU and DGB in the relation between NMP and SA during the COVID-19 pandemic lockdowns. The hypothetical conceptual model was presented in Figure 1.

### 3.2. Critical case and participants

The present study employed a critical case sampling strategy to determine the study group. Critical case sampling is a purposive sampling technique that is particularly beneficial in exploratory research as well as research with limited resources. It is also useful in situations where a single case or a small number of cases can provide decisive insights into the phenomenon under scrutiny. Thus, in the present study the rationale for employing critical case sampling is that the phenomena under investigation are regarded as in line with “it exists everywhere if it is here; if it is not here, it does not exist anywhere” (Patton, 2002, p. 237). Thus, the case selected in this study is embedded with rich resources, and it was considered that the phenomena under scrutiny exist everywhere if they exist at students in this top-notch secondary school in Istanbul. Therefore, data were collected from 427 secondary school students who attend one of the top three secondary schools in Türkiye, which accepts students with the highest score of 500 and those who are in the 0.01 percentile according to the 2021 Nationwide Central Placement Exam (LGS) results.

The critical case in this study is one of the most prestigious secondary schools in Türkiye. It offers an advanced level of education with tuition-free facilities and boarding possibilities. Its diverse student body is drawn from all around Türkiye. The academic and extracurricular activities provided by the school encourage students to become lifelong learners and develop self-regulating, creative and critical thinking skills that enable them to contribute to society in various areas. The academic program is trilingual (Turkish, German, English) with a university preparatory curriculum, spanning 5 years, including 1 year of German preparatory and 4 years of secondary school. Since 2000, the school has offered the German Abitur diploma, which allows successful students to attend almost any German, Austrian, or Swiss university in almost any major, and is recognized by several member states of the EU. The students in this school have consistently scored some of the highest marks in both the Abitur and Turkish university examinations. The school has been a pioneer in education for over 130 years and is well known in Türkiye for its achievements. In recognition of its excellence, it has been awarded ‘The Seal of Academic Excellence’ as a German international school by the German government. Regarding its notable alumni roster, the school has produced a substantial number of distinguished officials, encompassing three former prime ministers as well as a considerable array of ministers and high-level executives, throughout its historical timeline. Thus, the students in this school are expected to have developed increased self-regulating skills which will help them avoid distractions and addictive behaviors such as NMP and SA. However, depleted self-regulation capacity across the students will increase the likelihood of addictive SMTU and DGB, as well as NMP and SA. Therefore, the sample drawn from this case is critical and expected to explain the phenomena under investigation, namely the mediating role of SMTU and DGB in NMP and SA, which can be attributed to a rich source of data. If the phenomena exist in this school, it exists every secondary school, which will raise extensive public concern and require immediate action by policy makers, school boards, parents and researchers.

Prior to data collection, the participants were presented with an online informed consent that provided details on the research objectives, research team, data collection, data processing, and the measures in place to safeguard participant privacy. Additionally, prior to the commencement of the study, a letter of approval was granted by the Ethics Committee Review Board of the affiliated university. Therefore, the study was conducted in accordance with the guidelines outlined in the Declaration of Helsinki. All participants voluntarily completed the surveys, and no incentives were offered to encourage participation.

### 3.3. Data collection instruments

The data were collected through (I) Personal Information Form, (II) Smartphone Addiction Scale (SAS-SV) developed by Kwon et al. (2013), and (III) Nomophobia Questionnaire (NMP-Q) developed by Yildirim and Correia (2015).

#### 3.3.1. The personal information form

The form was developed by the researchers. It included open-ended questions to gather some demographics such as age, gender, grade level, and some other questions to assess problematic use of social media tools and digital games played by students. The number of social media tools they use and digital games they play was coded and transferred into continuous variables to serve as multiple mediators in the mediation model.

#### 3.3.2. Smartphone Addiction Scale

The SAS-Short Version is a self-report scale comprising of 10 items rated on a 6-point Likert scale. It was developed by Kwon et al. (2013) and adapted to the Turkish context by Noyan et al. (2015). In the adaptation study, the scale was administered to a group of 367 undergraduate students in Türkiye. Consistent with the original scale, the Turkish version also had 10 items loaded in one factor and weighted in 6-point Likert type items with response options ranging from 1 = “*strongly disagree*” to 6 = “*strongly agree*.” The internal consistency coefficient of Cronbach’s alpha was 0.87, indicating high reliability, and the test–retest reliability coefficient was estimated as 0.93. In this study, the items of the scale yielded a mean score of 2.69 and standard deviation of 1.02, with a high internal consistency coefficient of 0.86.

#### 3.3.3. Nomophobia questionnaire

The NMP-Q was developed by Yildirim and Correia in 2015. The scale consists of 20 items loaded on four dimensions. Each item is rated using a 7-point Likert scale that ranges from 1 (*strongly disagree*) to 7 (*totally agree*). These ratings are then summed to generate a total score, with higher scores indicating greater severity of NMP. Furthermore, the NMP-Q score can be interpreted in terms of the level of NMP exhibited, with scores ranging from 20 to 140. Specifically, a score of 20 corresponds to an absence of NMP, while scores between 21 and 59 indicate a mild level, scores between 60 and 99 indicate a moderate level, and scores of 100 or greater indicate a severe level of NMP (Yildirim and Correia, 2015). In the present study, the Cronbach’s Alpha (*α*) was estimated for each subscale as such *not being able to communicate α* = 0.92, *losing connectedness α* = 0.85, *not being able to access information α* = 0.84 and *giving up convenience α* = 0.75. Cronbach’s Alpha (*α*) coefficient for the scale total is *α* = 0.92, which indicates a high level of psychometric quality. The scale items were summed as *M* = 3.24, SD = 1.18, indicating a mild level NMP.

#### 3.3.4. Social media tools usage

In order to specify the participants’ SMTU frequency, the participants were invited to complete an open-ended question to identify their preferences about social media tools. Regarding the social media tools they use, the great majority of the participants (*n* = 357) reported that they use YouTube (83%). In the second place, 324 use WhatsApp (76%), and finally 278 reported that they use Instagram (65%).

#### 3.3.5. Digital gaming behavior

The researchers used an open-ended questionnaire to gather the participants’ digital gaming preferences. A significant number of students reported that they play “No games” (27%) at all. Regarding the games they play, 104 reported that they play Minecraft (24%), in the second place, 84 play Counter Strike (20%), and finally 75 reported that they play Among Us (18%).

### 3.4. Analytical strategy

In the present study, the procedures of parallel multiple mediators model were employed as suggested by Preacher and Hayes (2004). Thus, Hayes PROCESS macro was utilized to compute total, direct, and indirect effects, including both total and specific effects for each mediator, and perform significance tests using BC bootstrap procedures. There is a growing body of research that prefers bootstrap procedures over the Sobel test and other traditional procedures because they do not require the normality assumption of the distribution of the indirect effects, leading to more robust protection against type II error (Teixeira et al., 2010). Therefore, the study presents results for bootstrap tests using a resample procedure of 5,000 bias-corrected bootstrap samples with 95% confidence intervals.

The statistical analysis was carried out using IBM SPSS Statistics (Version 25). Pearson correlation coefficient was estimated to examine the associations among the key variables. A multiple linear regression model was utilized to explore the potential mediating roles of SMTU and DGB in the relationship between NMP and SA. To investigate the parallel multiple mediating effect, we used Hayes’s PROCESS macros version 4.1. A bias-corrected confidence interval (CI) of 95% was calculated based on 5,000 BC bootstrapping samples, and a *p* value <0.05 was considered statistically significant when the CI did not include zero (MacKinnon et al., 2002).

### 3.5. Common method bias

To assess whether there exists an inflation or deflation of the true correlation among observable variables, known as common method bias (CMB), in the current study, an exploratory factor analysis (EFA) was conducted following the recommendation of Harman’s single factor test (Collier, 2020). The analysis purported a six-factor solution, where all factors had eigenvalues greater than 1, and the first factor explained 15.096% of the total variance. As the percentage of variance explained by the first factor was lower than the critical value of 40% suggested by Podsakoff et al. (2003), it can be concluded that no CMB was observed in the present study.

## 4. Results

The participants consisted of 427 secondary school students who were receiving online courses during the COVID-19 home confinement. The participants were 128 females (30%) and 299 males (70%). 38 (8.9%) of the participants were 14 years old, 90 (21.1%) were 15 years old, 184 (43.1%) were 16 years old, 67 (15.7%) were 17 years old, 45 (10.5%) were 18 years old and 3 (0.7%) were 19 years old. The average age was 15.93. Regarding the grade levels of the participants, 79 of the participants (18.5%) were in the preparatory class, 173 (40.5%) were at the 9th grade, 99 (23.2%) were at the 10th grade, 58 (13.6%) were at the 11th grade and finally 18 of them (4.2%) were at the 12th grade. The participants reported that 56 (13%) use only 1 social media tool, 100 (23%) use 2, 130 (30%) use 3, 96 (22%) use 4, 38 (9%) use 5 and 12 (3%) use 6 social media tools. The average SMTU was reportedly 2.99. On the other hand, the participants reported that 117 (27%) do not play any digital games. 139 (32%) play at least one game, 80 (19%) two games, 53 (12%) three games, 18 (4%) 4 games, 25 (6%) 5 or more games. The average game play count was reportedly 1.55.

### 4.1. Preliminary analysis

Prior to investigating the multiple mediator role of SMTU and DGB, the means, standard deviations, Skewness-Kurtosis values, and correlation coefficients for key variables were estimated and presented in Table 1.

**Table 1:** Means, standard deviations, Skewness-Kurtosis, and correlations between the key variables.

Variables	*N*	*M*	*SD*	Skewness	Kurtosis	1	2	3	4
1. NMP	427	3,24	1,18	0.327	−0.405	1			
2. SMTU	427	2,99	1,26	0.229	−0.489	0.216**	1		
3. DGB	427	1,55	1,51	1,226	1,424	−0.002	0.162**	1	
4. SA	427	2,69	1,02	0.360	−0.528	0.623**	0.222**	0.045	1

*p < 0.05, **p < 0.01.

NMP: Nomophobia, SA: Smartphone Addiction, SMTU: Social Media Tools Usage, DGB: Digital Gaming Behavior.

Table 1 illustrates that all four correlations were statistically significant. NMP positively correlated with SMTU (*r* = 0.216, *p* < 0.01) and SA (*r* = 0.623, *p* < 0.01). Additionally, SMTU positively correlated with DGB (*r* = 0.162, *p* < 0.01) and SA (*r* = 0.222, *p* < 0.01). However, DGB was not significantly associated with other key variables. The skewness and Kurtosis values were found to be in the range of ±1.5, which ensured the normality assumptions (Tabachnick and Fidell, 2012).

### 4.2. The parallel multiple mediation analysis

**Figure 2:**
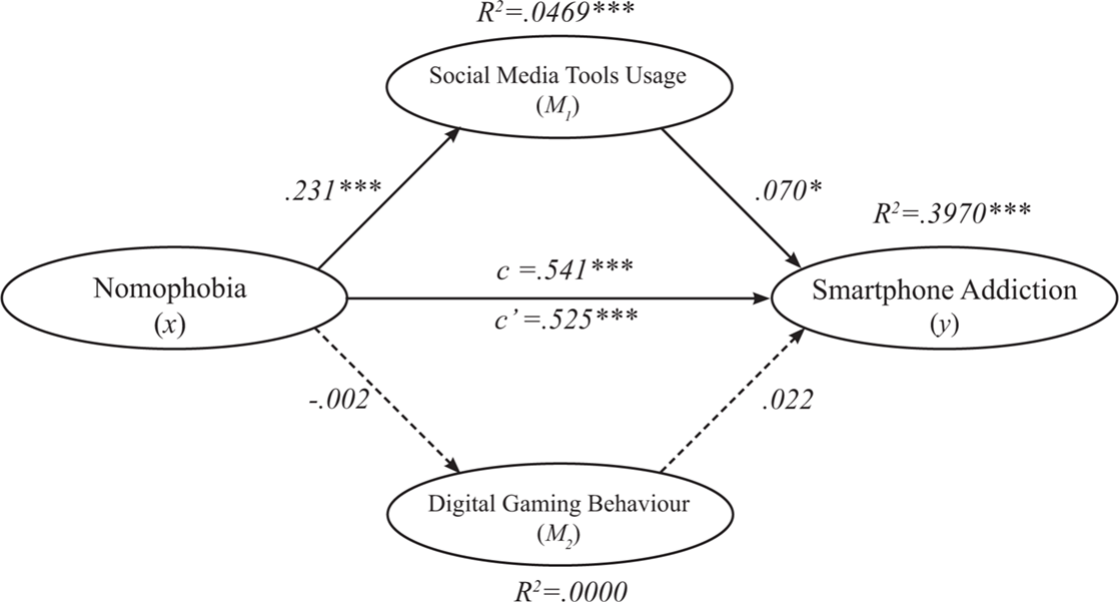
Parallel multiple mediator model.

There are two classifications of multiple mediator models. If the mediators are connected in a causal sequence, then it is called as the serial multiple mediator model. Alternatively, they are only associated with each other without causing any causal influence, it is called as the parallel multiple mediator model. Based on this distinction, a parallel multiple mediator model assumes that antecedent variable *x* impacts subsequent variable *y* both directly and indirectly through two or more mediators, on the condition that no mediator has a causal effect on any other mediator (Hayes, 2022). Given this, we employed a parallel multiple mediator model in order to test if NMP impacts SA both directly or indirectly through SMTU and DGB. The results were presented in Table 2 and Figure 2. In the multiple mediator analysis, first the significance of the relationship between NMP and SA (c path) was tested [*b* = 0.541, *t* = 16.512, *p* < 0.001 (0.4768 /0.6057)]. Thus, H1 in our study was supported. Then, the mediating role of SMTU and DGB in the relationship between secondary school students’ NMP and SA was tested by employing BC bootstrapping with 5,000 samples. As a second step, the relationship between NMP and SMTU (a1 path) was tested. The a1 path was found to be positive and significant [*b* = 0.231, *t* = 4.597, *p* < 0.001 (0.1323 / 0.3298)], supporting the second assumption and H2.1. Third, the relationship between NMP and DGB (a2 path) was tested. The a2 path was found to be negative and not significant [*b* = −0.002, *t* = −0.033, *p* = 0.973 (−0.1237 / 0.1196)], not supporting the third assumption and H2.2. Next, the relationship between SMTU and SA was tested (b1 path), which was found to be positive and significant [*b* = 0.070, *t* = 2.199, *p* < 0.05 (0.0074 / 0.1322)]. This also supported the fourth assumption and H3.1. Later, the relationship between DGB and SA was tested (b2 path), which was found to be positive but not significant [*b* = 0.022, *t* = 0.8446, *p* = 0.398 (−0.0289 / 0.0724)]. This finding did not support the fifth assumption and H3.2. The results also indicated that NMP has a significant indirect effect on the SA through SMTU. Thus, the mediating role of the SMTU was found to be positive and significant, supporting the sixth assumption and H4.1. However, the findings demonstrated that NMP did not have a significant effect on SA through DGB. Thus, the mediating role of DGB was found to be nonsignificant, not supporting the seventh assumption and H4.2. Additionally, the direct effect of NMP on SA (c’ path) became weaker but still significant in the presence of the mediator variable SMTU [*b* = 0.525, *t* = 15,709, *p* < 0.001 (0.4594 /0.5909)]. This is an indicator of SMTU playing a partial mediating role. As a conclusion, SMTU partially mediated the relationship between NMP and SA. The results of the mediation analysis were introduced in Table 2 and Figure 2.

**Table 2:** Summary of mediation analysis.

Hypothesized relationships	Direct effect	Indirect effect	*t*	95% CI		Value of *p*	Conclusion
				*LLCI*	*ULCI*		
H1: NMP → SA	0.541***		16.512	0.4768	0.6057	0.000	H1 Supported***
H2.1: NMP → SMTU	0.231**		4.597	0.1323	0.3298	0.000	H2.1 Supported***
H2.2: NMP → DGB	−0.002		−0.033	−0.1237	0.1196	0.973	H2.2 Not Supported
H3.1: SMTU → SA	0.070*		2.199	0.0074	0.1322	0.028*	H3.1 Supported*
H3.2: DGB → SA	0.022		0.8446	−0.0289	0.0724	0.398	H3.2 Not Supported
H4: NMP → SA	0.525***		15.709	0.4594	0.5909	0.000	H4.1 Supported *** H4.2 Not Supported
NMP**→**							
SMTU**→**		0.0161		0.0023	0.0033		H4.1: Partial Mediation
DGB**→**		−0.0000		−0.0042	0.0045		H4.2: No Mediation
SA							
Squared multiple correlations (*R*^2^)							
SMTU	0.0469	*F* (1, 430) = 21.139, *p* = 0.000					
DGB	0.0000	*F* (1, 430) = 0.011, *p* = 0.973					
SA	0.3970	*F* (3, 428) = 93.919, *p* = 0.000					

*p < 0.05, **p < 0.01, ***p < 0.001.

NMP: Nomophobia, SA: Smartphone Addiction, SMTU: Social Media Tools Usage, DGB: Digital Gaming Behavior.

Unstandardized coefficients were reported. BC Bootstrap with 5,000 samples and 95% CI.

## 5. Discussion and conclusion

Adolescents are increasingly affected by the developments in digital technologies, which has become more prominent with the outbreak of the COVID-19 pandemic. The pandemic enforced some infection control measures such as home confinement and lockdowns, leading adolescents to spend more time at home and rely heavily on ICT tools, which caused adverse psychological and behavioral effects (Serra et al., 2021). In particular, online remote teaching practices during the pandemic limited adolescents’ socialization opportunities, leading to a substantial upsurge in digital gaming behaviors (DGB) and addictive social media tools usage (SMTU) through smartphones. However, excessive use might lead to addictive tendencies including smartphone addiction (SA), and nomophobia (NMP). Although there is an empirical gap since few research studies have focused on the underlying mechanism among these variables, previous research results provided some implications about SMTU and DGB might have a mediating role in this relation. Hence, the present study aimed at exploring the parallel mediating role of secondary school students’ SMTU and DGB in the relationship between NMP and SA.

To our knowledge, the present study is the first to explore the relationship between NMP and SA amidst the COVID-19 home confinement and the parallel mediating role of DGB and SMTU in this relation. Our findings revealed that NMP is positive predictor of SA and SMTU. However, no significant associations were found between NMP and DGB. SMTU is positively correlated with SA, and contrary to assumptions, no significant associations were found between DGB and SA. As for the mediating roles, SMTU partially mediates the relation between NMP and SA, however the mediating role of DGB on NMP and SA is not significant. Details about the findings were presented in the following sections.

### 5.1. NMP is positively associated with SA

The results of the correlational analysis and mediating analysis revealed that NMP was positively associated with SA (H1 supported) (*b* = 0.541, *t* = 16.512, *p* < 0.001 [0.4768 /0.6057]). This finding is concurrent with some previous research findings, indicating that during lockdowns smartphone use and NMP increased dramatically (Serra et al., 2021; Shahzad et al., 2021). Smartphones provide many conveniences to students and teachers in the education process (Almunawar et al., 2015). Despite the conveniences provided by smartphones which are a source of entertainment and spending time apart from educational purposes, heavily reliance on smartphones have increased students’ NMP levels and led to an increase in students’ SA (Shahzad et al., 2021). This finding was supported by our results. Yet even before the pandemic, one out of every three students has a problem with NMP (Tavolacci et al., 2015). It has been revealed that the COVID-19 pandemic prevents students’ social interactions and increases SA and SMTU, causing NMP (Pasquale et al., 2021). Our findings also provided some empirical evidence for previous research studies asserting that there is a strong link between NMP and SA (Tavolacci et al., 2015; Anshari et al., 2019; Durak, 2019; Semerci, 2019).

### 5.2. NMP is positively associated with SMTU

The findings of the present study also indicated that there was a positive significant relationship between NMP and SMTU (H2.1 supported) [*b* = 0.231, *t* = 4.597, *p* < 0.001 (0.1323 / 0.3298)]. This finding mostly overlapped with the previous research in the literature in that adolescents mostly use social media and play digital games through smartphones, which makes them vulnerable to addictive behavior and this might cause NMP amongst them (Yildirim and Correia, 2015) and there is an interaction between NMP and addictive SMTU (Lin et al., 2021). Additionally, previous research highlighted that adolescents perceive social media as safe places to express themselves freely (Shah et al., 2019). However, excessive use of social media can cause negative consequences such as NMP (Bergagna and Tartaglia, 2018; Lin et al., 2021). In a similar vein, excessive social media use increased the risk of social isolation and loneliness in adolescents, thus leading to NMP (Ciacchini et al., 2023). Our results mostly concurred with the previous research findings, which illustrated that there is positive association between NMP and SMTU. Extracted from the results, it is advisable that healthcare providers, educators, parents, and other relevant stakeholders should pay more heed to the issues of NMP and addictive SMTU among adolescents. These issues are of particular concern because they may lead to sleep problems among adolescents, which can have negative impacts on their overall well-being over time (Lin et al., 2021). Therefore, it is important to gain a deeper understanding of nomophobia and social media addiction in order to develop effective interventions to address these issues and promote healthy digital behaviors across secondary school students.

### 5.3. NMP is not significantly associated with DGB

Contrary to the findings of previous research, the present study did not find a significant relationship between NMP and excessive DGB, which was somewhat surprising (H2.2 not supported). Previous studies have suggested that excessive DGB can lead to psychological problems and increase the risk of NMP. However, the findings of the current study did not provide empirical support for these claims. For instance, Lee (2014) found that adolescents’ frequent use of smartphones for social media and gaming makes them susceptible to NMP. In a similar vein, Sandeep (2018) argued that excessive use of digital gaming may lead to NMP. Nonetheless, the present study’s results did not overlap with the findings of these studies. On the other hand, the addictive gaming literature has yielded conflicting results. For example, during the COVID-19 pandemic, digital gaming was found to be a helpful tool for managing stress and mitigating the negative effects of public health measures such as lockdowns, quarantines, and social distancing (Balhara et al., 2020). Therefore, it is possible that participants in the current study might have experienced some positive effects of digital gaming during the COVID-19 home confinement and lockdowns. Alternatively, excessive digital gaming might have had a function as a coping mechanism for addressing shortcomings of COVID-19 lockdowns as suggested by previous research (Griffiths and Beranuy, 2009). The issue of online gaming addiction is a real-world problem that needs practical and effective solutions. However, the development of such solutions is hindered by the limited scope of research on the psychological, sociological, and physiological effects of addictive DGB. To address this limitation, there is a need for a more comprehensive and well-established body of literature that can inform education, prevention, intervention, treatment, and legislative policy regarding online gaming addiction (Griffiths, 2009). Therefore, more research is needed to provide educators and other stakeholders with evidence-based policy decisions to make a distinction between excessive and addictive DGB.

### 5.4. SMTU is positively associated with SA

This study found a significant positive association between SMTU and SA (H3.1 supported) [*b* = 0.070, *t* = 2.199, *p* < 0.05 (0.0074 / 0.1322)]. This finding mostly concurred with the literature. Overuse of the internet, reliance on smartphones, and addictive use of SMTU and digital games are prevalent among adolescents. This situation can make adolescents more vulnerable to SA (Valkenburg and Peter, 2011). Additionally, research illustrates that excessive smartphone use might have negative effects on mental health, cognitive functioning, and sleep quality (Lin et al., 2021). Research also purported that there is a positive correlation between time spent on social media and SA (Marengo et al., 2022). On the other side, previous research argued that SA significantly predicted SMTU (Savcı and Aysan, 2017), which illustrates the complex interplay between SMTU and SA. However, it is well documented in the literature that the most common problems that many adolescents currently experience are SA, and reliance on SMTU and DGB (Yiğit and Günüç, 2020). Our findings are consistent with these previous studies, highlighting the need for in-depth exploration of the complex relationship among these variables. Yet the literature on the impact of social media on adolescents yields conflicting findings, indicating that its effects on psychological outcomes can be both positive and negative (Billieux et al., 2015). This suggests that young people experience a paradox in relation to social connectedness through online tools. While these tools enable individuals to form and join online groups and communities with greater ease, they can also lead to feelings of alienation and ostracism as documented by a review study (Allen et al., 2014). Thus, the increasing prevalence of SMTU among adolescents necessitates a thorough understanding of its potential positive and negative impacts. Therefore, teachers, parents, and school administrators should take prompt action to address these issues and improve the quality of life and academic performance of adolescents.

### 5.5. DGB is not significantly associated with SA

The findings, somewhat surprisingly, illustrated that there was not a significant relationship between DGB and SA (H3.2 not supported). Contrary to assumptions and previous research findings, the study disclosed that there was no significant associations between DGB and SA (b2 path) [*b* = 0.022, *t* = 0.8446, *p* = 0.398 (−0.0289 / 0.0724)]. Thus, this finding was not congruent with the literature. Research illustrated that smartphones are regarded as the most important digital gaming and social media tools amongst adolescents (Yildirim and Correia, 2015). Smartphone addiction, digital gaming and social media use are the most common problems amongst adolescents (Yiğit and Günüç, 2020). In this sense, problematic gaming behavior among the adolescents may include the inability to stop playing for an extended period, confusion between the game and reality, neglection of responsibilities due to excessive gaming, and a preference for gaming over other activities (Horzum, 2011), insomnia and poor quality of life (Fazeli et al., 2020). In this context, digital game players are more likely to display behavioral addiction (Grüsser et al., 2007; Griffiths, 2010; Kuss and Griffiths, 2012). Additionally, Savcı and Aysan (2017) argued that digital gaming and SA can anticipate SMTU. Likewise, Liu et al. (2016) found that there was a significant association between smartphone gaming, frequent smartphone use, and SA. Specifically, both the group of individuals who predominantly use their smartphones for gaming and those who use multiple applications on their smartphones for gaming displayed a similar association with SA. Yet these findings were not supported in the present study. However, it is worth noting that the literature on addictive gaming has produced conflicting findings. For instance, recent research has suggested that digital gaming may serve as a helpful tool for managing stress and mitigating the negative effects of COVID-19 (Balhara et al., 2020). In a similar vein, Griffiths and Beranuy (2009) asserted that excessive digital gaming may serve as a coping mechanism for addressing personal shortcomings or difficulties such as a lack of social support, relationship issues, or dissatisfaction with one’s appearance. Similar findings were documented in research studies indicating that playing digital games can offer potential benefits, including cognitive, motivational, emotional, and social benefits (Granic et al., 2014), and alleviating feelings of isolation (Carras et al., 2017). In this sense, it is likely that the participants in the present study may have experienced some positive impacts of digital gaming during the COVID-19 home confinement and lockdowns. Furthermore, they may have employed digital gaming as a coping mechanism to mitigate the shortcomings or difficulties of COVID-19 lockdowns. Thus, more research is needed to gain a comprehensive understanding of the complex interplay between digital gaming and smartphone addiction, particularly in the context of the COVID-19 pandemic or future crisis.

### 5.6. SMTU mediates the relation between NMP and SA

In this study, the mediating role of SMTU in the relation between NMP and SA was confirmed (H4.1 supported). Additionally, the direct effect of NMP on SA (c’ path) became weaker in the presence of the mediator variable SMTU (*b* = 0.525, *t* = 15,709, *p* < 0.001, [0.4594 /0.5909]). This means adolescents’ level of NMP and SA was partially influenced by SMTU. We can conclude from the findings that SMTU is a significant positive predictor of NMP and SA on adolescents. Thus, adolescents who exhibit excessive SMTU are more likely to experience NMP and SA. Given the significant negative effects of NMP and SA on adolescents’ well-being, it is notable to pay some heed to SMTU and its potential negative impacts on mental health. Thus, it is crucial to draw attention to the issue of SMTU as a factor that could potentially lead to adverse outcomes for adolescents. The negative effects of addictive SMTU have raised concerns among adolescents and others, including mental distress, depression, sleep disruption, and insomnia (OECD, 2017). In line with this, previous research has documented a range of negative consequences associated with NMP and SA, such as psychological distress, depression, anxiety, sleep disturbances, unhealthy habits, and reduced academic success (Brunborg et al., 2014; Carey et al., 2021), social detachment, anxiousness, worry about contagion, depression and insomnia (Huang and Zhao, 2020; Qiu et al., 2020). These findings underscore the importance of addressing SMTU to mitigate the negative effects of NMP and SA in adolescents. Therefore, school boards and researchers should pay heed to mediating role of SMTU in the development of effective interventions such as cognitive-behavioral therapy, and coping strategies, which will help students develop healthier digital habits and avoid excessive SMTU. These interventions in turn may prevent the negative outcomes associated with NMP and SA across secondary school students. Additionally, school boards and researchers may consider implementing programs aimed at raising awareness about the potential risks of SMTU, leading to exacerbation of NMP and SA.

### 5.7. DGB does not mediate the relation between NMP and SA

In this study, the mediating role of DGB in the relation between NMP and SA was not confirmed, and NMP was not associated with SA in the presence of DGB as a mediator (H4.2 not supported). Although most of the findings of current study were consistent with existing literature it was surprising to some extent that there was no direct effect of NMP on SA in the presence of DGB. Adolescents level of NMP and SA was not influenced by DGB. This can be an indicator of that adolescents’ performing excessive DGB does not lead to NMP and SA. We can conclude from the above findings that DGB is not a significant predictor of SA in adolescents. This result can be attributable to the special characteristics of the participants because they are the students who got the highest score of 500 and ranked in the 0.01 percentile according to the 2021 Nationwide Central Placement Exam. As they have a high academic orientation and achievement motivation, they can simply self-regulate their own addictive tendencies or employ digital gaming as a coping strategy to mitigate the negative impacts of COVID-19 lockdowns. Therefore, further research is needed to explore the potential mediating role of DGB in the relationship between NMP and SA, especially with diverse sample groups. This would not only help to confirm the current research findings but also provide a better understanding of the conflicting results in the literature regarding the positive or and negative outcomes of DGB. In addition, such studies could help to identify potential intervention strategies for addressing the addictive digital gaming and SA as well as their associated negative impacts on mental health.

As a conclusion, the COVID-19 pandemic and resulting home confinements and lockdowns have led to an increase in excessive technology use among secondary school students. This has raised some concerns regarding NMP, DGB, SMTU, SA, and associated behavioral disorders and technological addiction. The present study documented some empirical evidence indicating the links between NMP and SA, NMP and SMTU, SMTU and SA across secondary school students. Surprisingly, no significant associations were found between NMP and DGB or between DGB and SA. Furthermore, the study proved the partial mediating role of SMTU in the relation between NMP and SA. However, no mediation was found for DGB. The study contributes to a better understanding of the complex interplay among NMP, SA, DGB, and SMTU as well as their prevalence during COVID-19 home confinement. The study provided some implications and recommendations for parents, teachers, schools, and adolescents to address the negative impacts of NMP, SA, DGB, and SMTU.

## 6. Implications and limitations

The contribution of this research has a bilateral impact on research community both theoretical and practical sides. Regarding theoretical contributions, previous research has focused on the study of the associations between NMP and SA, NMP and DGB, NMP and SMTU and so forth. However, the present study provides some empirical evidence to explain the complex interplay between these variables in addition to highlighting the parallel mediating role of SMTU and DGB in the relation between NMP and SA. More specifically, our results are robust and contribute to a better understanding of underlying complex mechanism in this relation. Regarding practical contributions, it helps public understanding of addictive SMTU, DGB, NMP and SA during the COVID-19 pandemic which have brought about behavioral disorders and technological addiction across secondary school students. More specifically, the implications of our findings are significant for school administrators, teachers, and researchers, as they highlight the need for the development of effective intervention programs and classroom guidance initiatives aimed at assisting students who struggle with NMP and SA. These programs could be devised to address the underlying causes of smartphone addiction and nomophobia, promote healthy digital behaviors, and provide support for affected students. By implementing such programs, school systems and educational institutions can play an active role in promoting the mental health and well-being of their students, while also enhancing academic performance and fostering a positive learning environment.

In addition to its key contributions, the present study provides some practical implications for society, as it sheds light on the psychological and sociological effects of social media and digital gaming on the psychosocial well-being and mental health of adolescents. The study also raises awareness about the growing public concern regarding addictive SMTU. Interestingly, our findings revealed that unlike SMTU, DGB was not significantly associated with NMP and SA. These results provide parsimonious support for previous studies highlighting the potential benefits of digital gaming (Griffiths and Beranuy, 2009; Balhara et al., 2020). By increasing public awareness of these issues, our study may contribute to the development of more effective school guidance and counseling services and interventions aimed at promoting healthy digital behaviors and supporting the mental health and well-being of secondary school students.

Despite its several contributions, a few limitations of the study are inherent and should be acknowledged. First, the sample is restricted to Turkish adolescents studying in one of the most prestigious secondary schools with a highest 0.01 percentile according to the Nationwide Central Placement Exam results. Thus, critical case sampling employed in the present study calls for some caution regarding the generalizability of the results to other populations. Second, the study utilized a cross-sectional design, and relied on self-reported data, which could introduce common method bias (CMB). Therefore, there is a possibility of CMB in this study although we have tested for and no CMB was traced, it is still possible that the relationships between variables may have been over or underestimated. Thus, it was not possible to establish causal relationships between the variables under investigation. These biases are bound to exist and cannot be avoided in survey designs.

Overall, the current research presented valuable empirical support for the previous research studies and contributed a better understanding of the interplay between NMP, SMTU, DGB, and SA. Still, there is a need for more research studies to provide a clearer picture of the phenomena under scrutiny. Therefore, future research may use longitudinal designs to further test the mediation model and reduce the potential for biases. Furthermore, future research should include larger and more diverse samples from various schools and cities and also can employ qualitative or mixed method research design to gain a more comprehensive understanding of the complex interplay among the variables.

## Data availability statement

The raw data supporting the conclusions of this article will be made available by the authors, without undue reservation.

## Ethics statement

The studies involving human participants were reviewed and approved by Hitit University Non-Interventional Studies Ethical Board. Written informed consent to participate in this study was provided by the participants’ legal guardian/next of kin.

## Author contributions

All authors listed have made a substantial, direct, and intellectual contribution to the work and approved it for publication.

## Conflict of interest

The authors declare that the research was conducted in the absence of any commercial or financial relationships that could be construed as a potential conflict of interest.

## Publisher’s note

All claims expressed in this article are solely those of the authors and do not necessarily represent those of their affiliated organizations, or those of the publisher, the editors and the reviewers. Any product that may be evaluated in this article, or claim that may be made by its manufacturer, is not guaranteed or endorsed by the publisher.
